# Supplementary material for: Machine Learning-Based Analysis of Glioma Grades Reveals Co-Enrichment
Source: Cancers (Basel). 2022 Feb 17;14(4):1014. doi: 10.3390/cancers14041014 (PMC8870250; doi:10.3390/cancers14041014)
Supplement: Supplementary file 1 [file cancers-14-01014-s001.zip › supplementaryMaterials.pdf]

Supplementary Materials

Machine learning-based analysis of glioma grades reveals co-enrichment

Mateusz Garbulowski[1,2,*], Karolina Smolinska[1], Uğur Çabuk[1,3,4], Sara A. Yones[1], Ludovica Celli[1,5,6], Esma Nur Yaz[1,7], Fredrik Barrenäs[1,8], Klev Diamanti[1,9], Claes Wadelius[9] and Jan Komorowski[1,8,10,11,*]

[1]Department of Cell and Molecular Biology, Uppsala University, Uppsala, Sweden

[2]Department of Biochemistry and Biophysics, Science for Life Laboratory, Stockholm University, Solna, Sweden

[3]Polar Terrestrial Environmental Systems, Alfred Wegener Institute Helmholtz Centre for Polar and Marine Research, Potsdam, Germany

[4]Institute of Biochemistry and Biology, University of Potsdam, Potsdam, Germany

[5]Institute of Molecular Genetics Luigi Luca Cavalli-Sforza, National Research Council, Pavia, Italy

[6]Department of Biology and Biotechnology, University of Pavia, Pavia Italy

[7]Department of Biomedical Engineering and Bioinformatics, The Graduate School of Engineering and Natural Sciences, Istanbul Medipol University, Istanbul, Turkey

[8]Washington National Primate Research Center, Seattle, WA, USA

[9]Department of Immunology, Genetics and Pathology, Uppsala University, Uppsala, Sweden

[10]Swedish Collegium for Advanced Study, Uppsala, Uppsala, Sweden

[11]Institute of Computer Science, Polish Academy of Sciences, Warsaw, Poland

*Correspondence: M.G. mateuszgarbulowski@gmail.com; J.K. jan.komorowski@icm.uu.se

Table of Contents

Supplementary Figures 3

Supplementary Captions for Tables S1-S8..25

Supplementary References for Table S1..26

Supplementary Figures

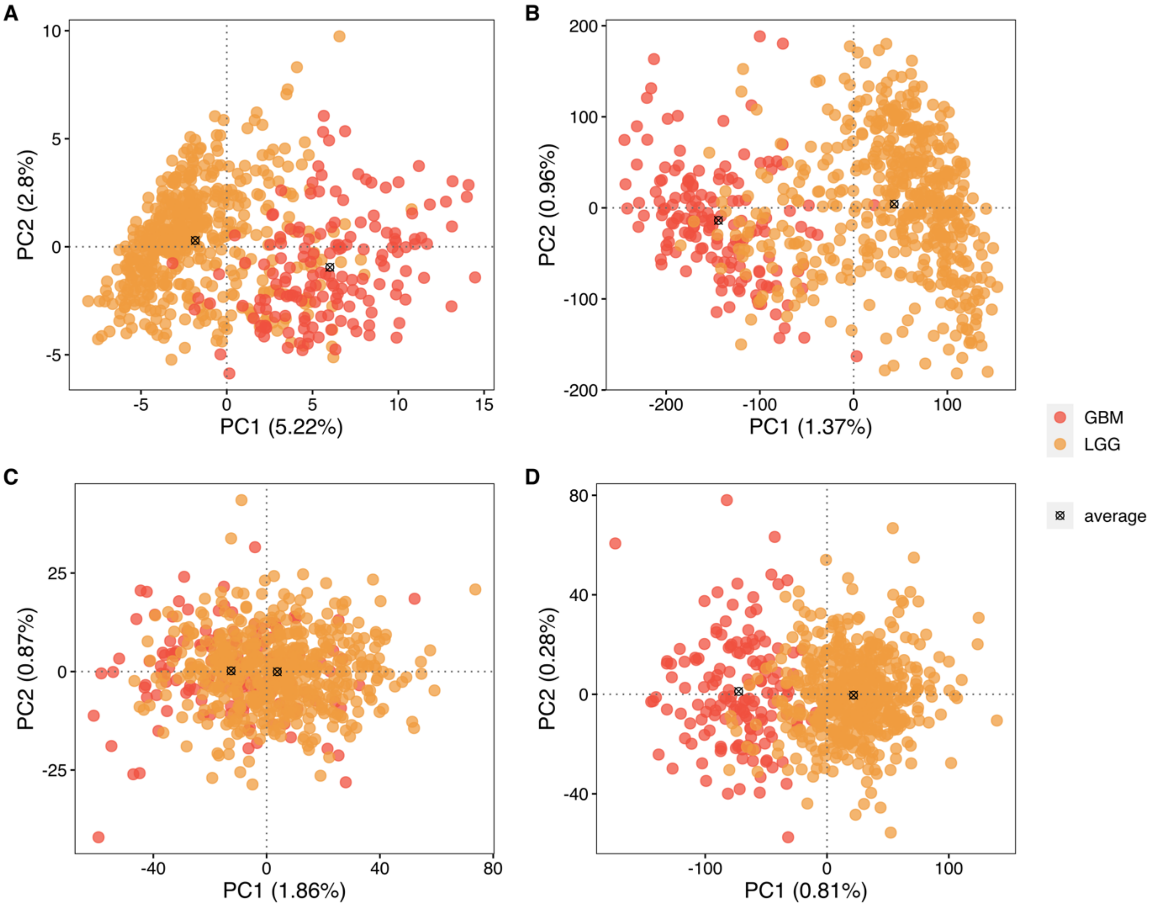

Figure S1. Principal component analysis (PCA) to investigate the variance for The Cancer Genome Atlas (TCGA) cohorts for Lower Grade Glioma (LGG) and Glioblastoma Multiforme (GBM). (**A**) Original data from the GDC repository. (**B**) Unified cohorts from the UCSC Xena repository. (**C**) Batch effect correction of unified cohorts for the *leek* parameter with 532 surrogate variables. (**D**) Batch effect correction of unified cohorts for the *be* parameter with 48 surrogate variables.

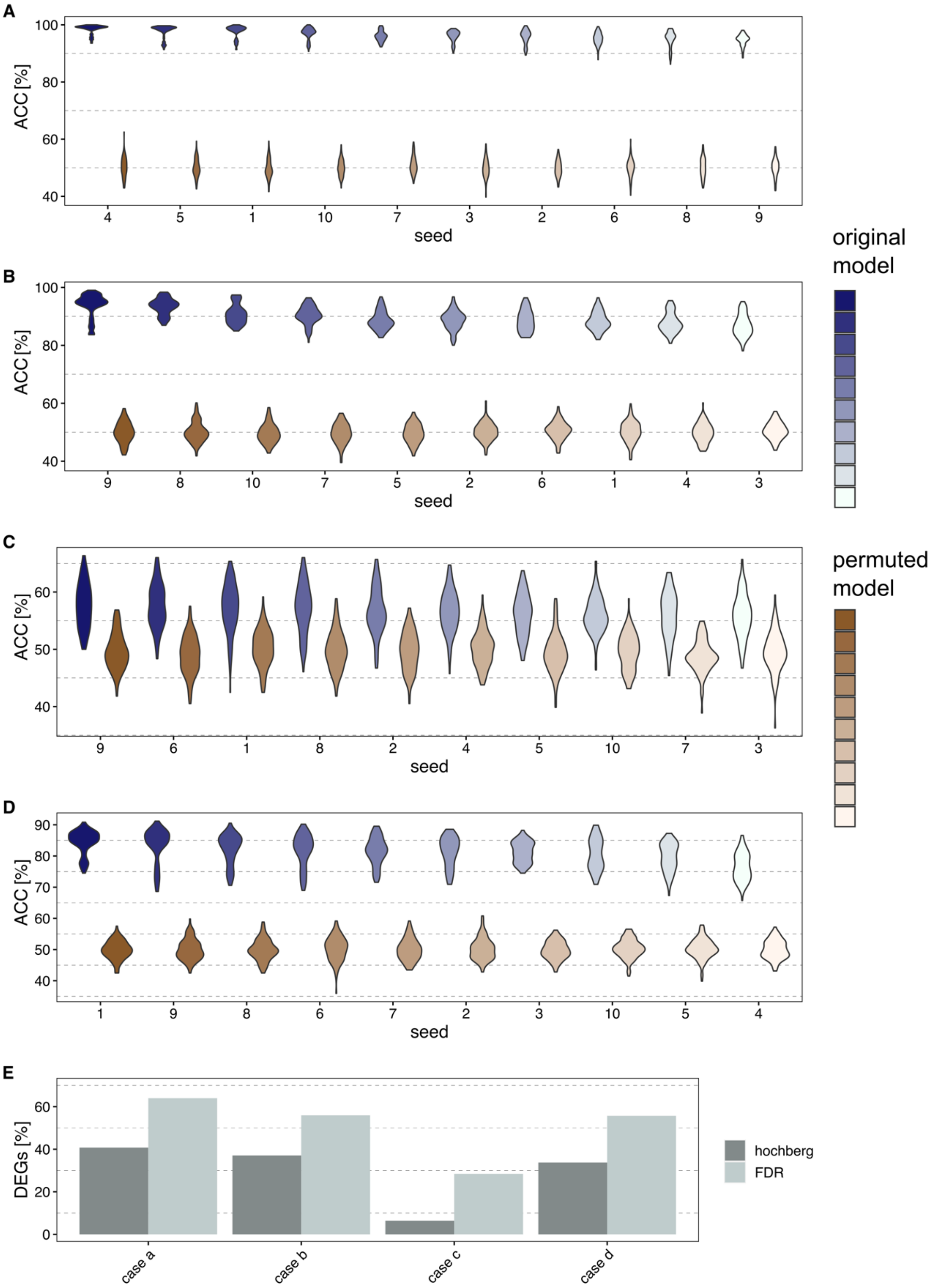

Figure S2. Machine learning analysis was performed on 100 randomly selected genes with 10 various seed values to investigate the bias for TCGA cohorts of LGGs and GBMs. All five machine learning methods were applied, viz. SMO, IBk, Bagging, J48 and JRip (see section ML evaluation). (**A**) Original data from the GDC repository. (**B**) Unified cohorts from the UCSC Xena repository. (**C**) Batch effect correction of unified cohorts for the *leek* parameter with 532 surrogate variables. (**D**) Batch effect correction of unified cohorts for the *be* parameter with 48 surrogate variables. (**E**) Student's *t*-test results for particular cases a-d corresponding to subplots (**A-D**).

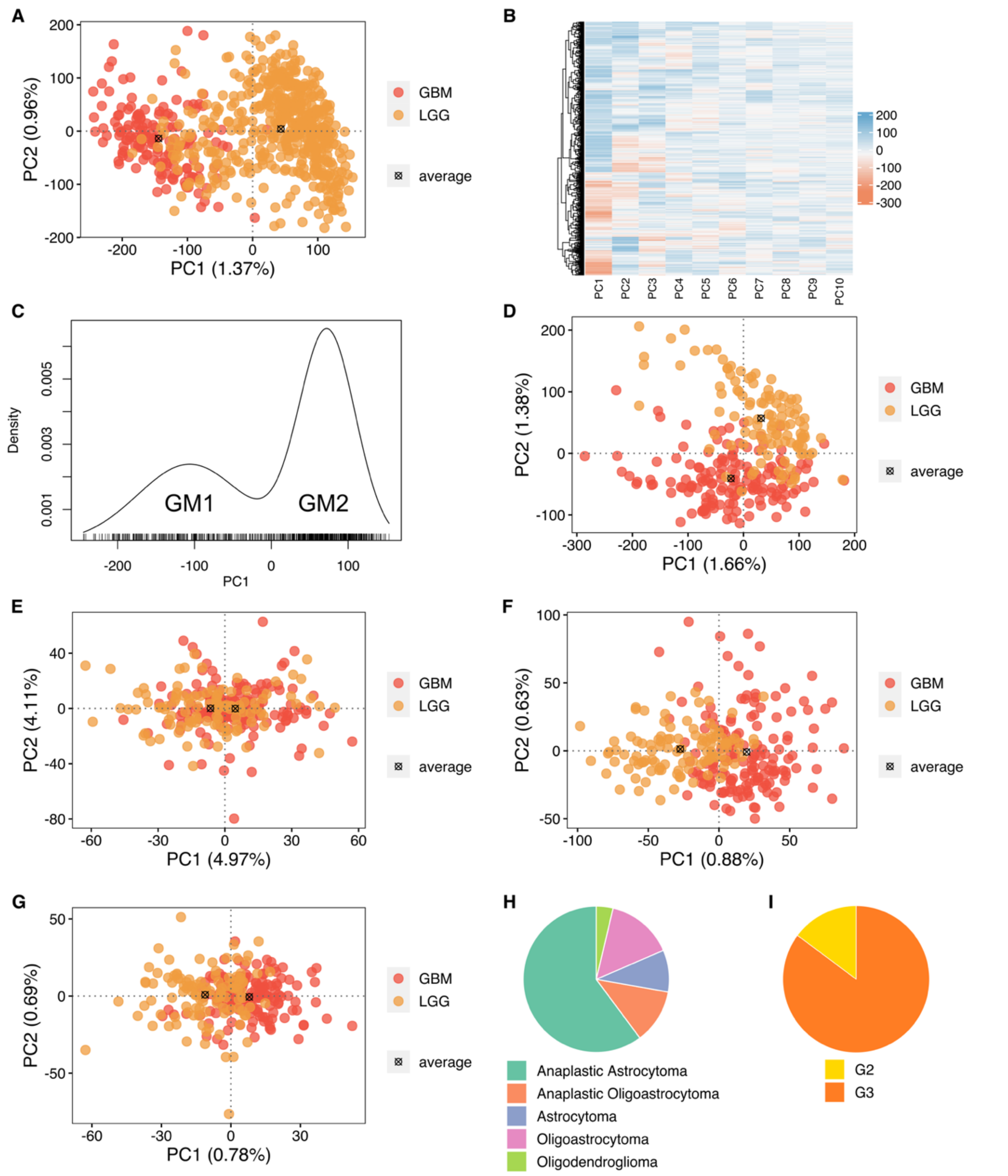

Figure S3. Data evaluation to localize and remove bias from the glioma cohorts (**A**) PCA for unified cohorts from the UCSC Xena repository. (**B**) Clustering of 1-10 PCs of unified cohorts from the UCSC Xena repository. (**C**) Gaussian mixtures (GM) were detected from PC1. In GM2-based sample sets, two GBMs were included in the mixture and excluded in further analyses. (**D**) PCA was performed on LGG and GBM samples selected based on GM1. (**E**) Batch effect correction performed on LGG and GBM samples selected based on GM1 for the *leek* parameter with 232 surrogate variables. (**F**) Batch effect correction for the *be* parameter with 30 surrogate variables performed on samples selected based on GM1. (**G**) The final data set was based on GM1 after batch effect correction and filtration for protein-coding genes (**H-I**) Evaluation of LGG samples included in GM1 based data set.

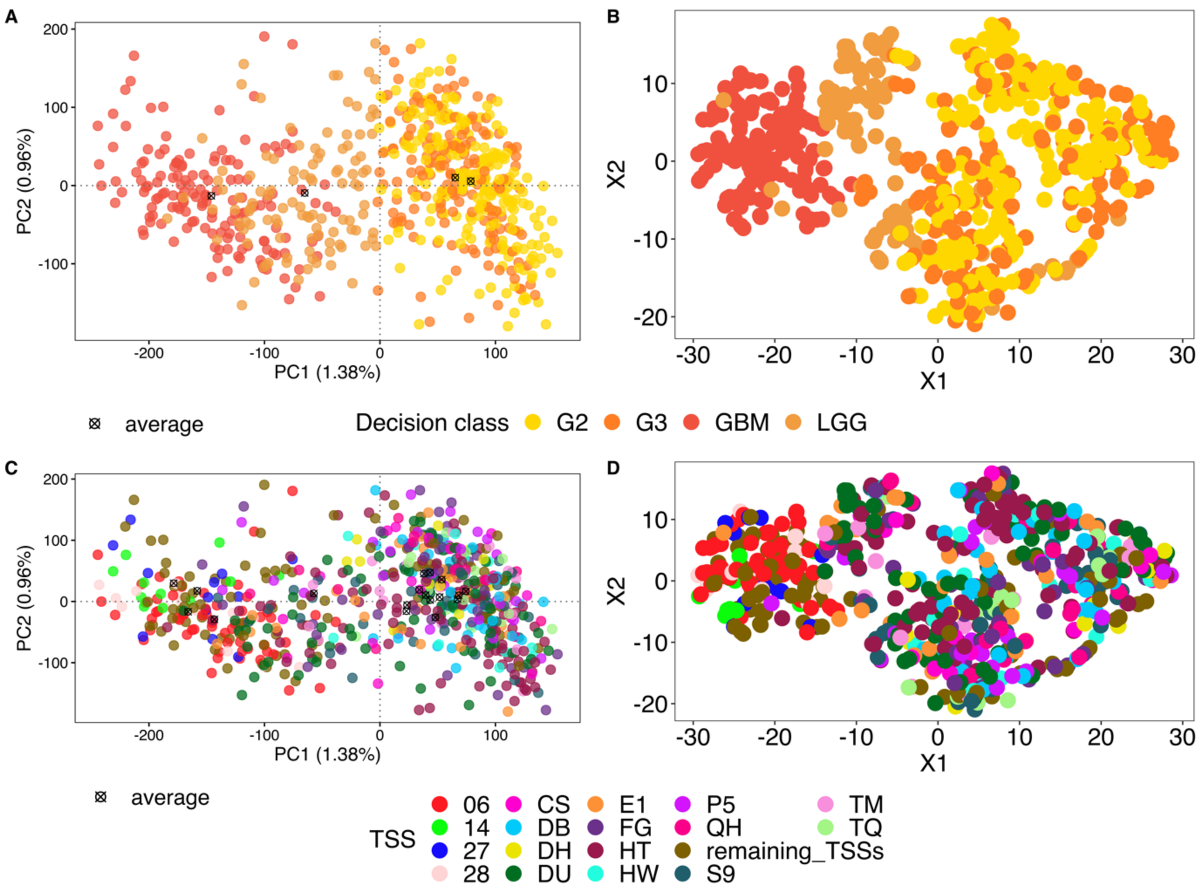

Figure S4. The global and local analysis of the data structure for TCGA glioma cohorts. (**A**) PCA and (**B**) t-SNE for decision class selected based on GMs. (**C**) PCA and (**D**) t-SNE for tissue source sites (TSSs). t-SNE plots were created for seed 1. Remaining TSSs included groups of samples equal to or less than 10.

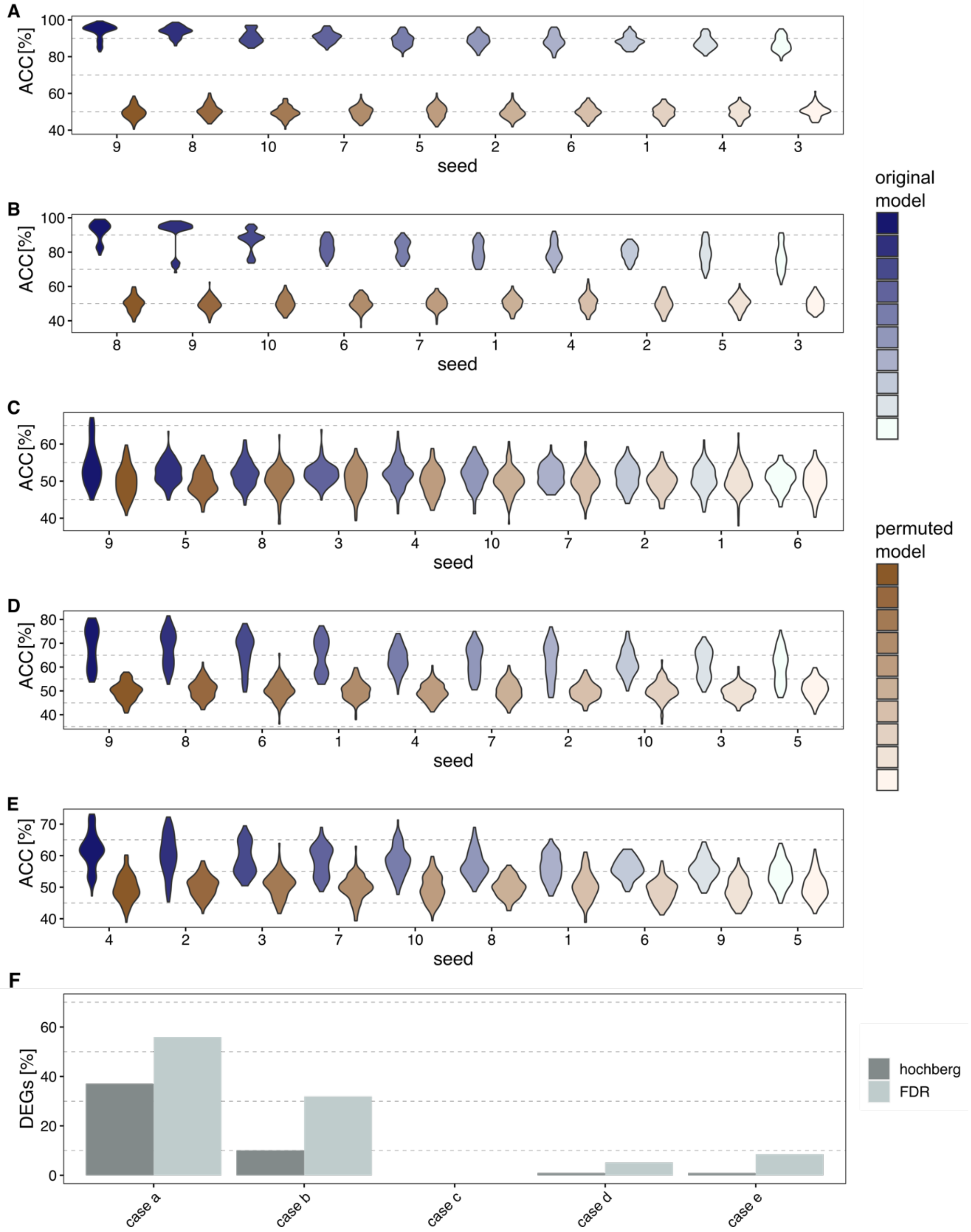

Figure S5. Machine learning analysis was performed on 100 randomly selected genes with 10 various seed values to investigate the bias for TCGA cohorts of LGGs and GBMs. All five machine learning methods were applied, viz. SMO, IBk, Bagging, J48 and JRip (see section ML evaluation). (**A**) unified cohorts from the UCSC Xena repository. (**B**) GM2 samples from unified cohorts from the UCSC Xena repository. (**C**) Batch effect correction of 259 GM2-based samples for the *leek* parameter with 232 surrogate variables. (**D**) Batch effect correction of 259 GM2-based samples for the *be* parameter with 30 surrogate variables. (**E**) The final data set is based on GM1 after batch effect correction and filtration for protein-coding genes. (**F**) Student's *t*-test results for particular cases a-e corresponding to subplots (**A-E**).

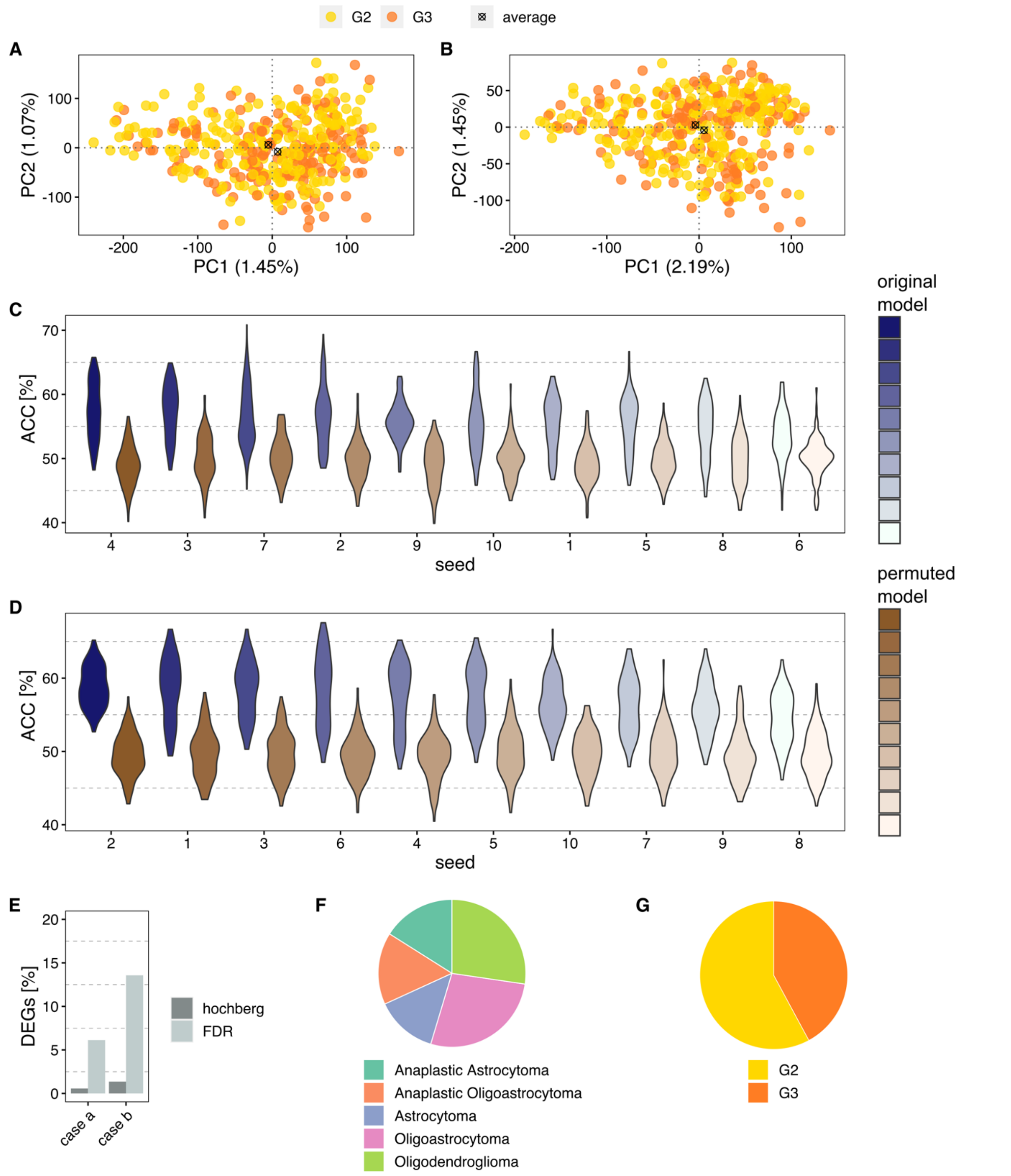

Figure S6. The evaluation of samples in GM2 consisting of the vast majority of LGGs. (**A**) PCA for LGGs based on GM2 samples. (**B**) PCA for LGGs based on GM2 samples for protein-coding genes only. (**C**) Machine learning evaluation of GM2 samples for discerning between GII and GIII. (**D**) Machine learning evaluation of GM2 samples for discerning GII and GIII for protein-coding genes only. (**E**) Evaluation of fraction of statistically significant genes discerning between GII and GIII with all genes (case a) and protein-coding genes only (case b). (**F**) The proportion of grades within a GM2-based data set for LGGs. For machine learning evaluation (**C**, **D**) all five methods were applied, viz. SMO, IBk, Bagging, J48 and JRip (see section ML evaluation).

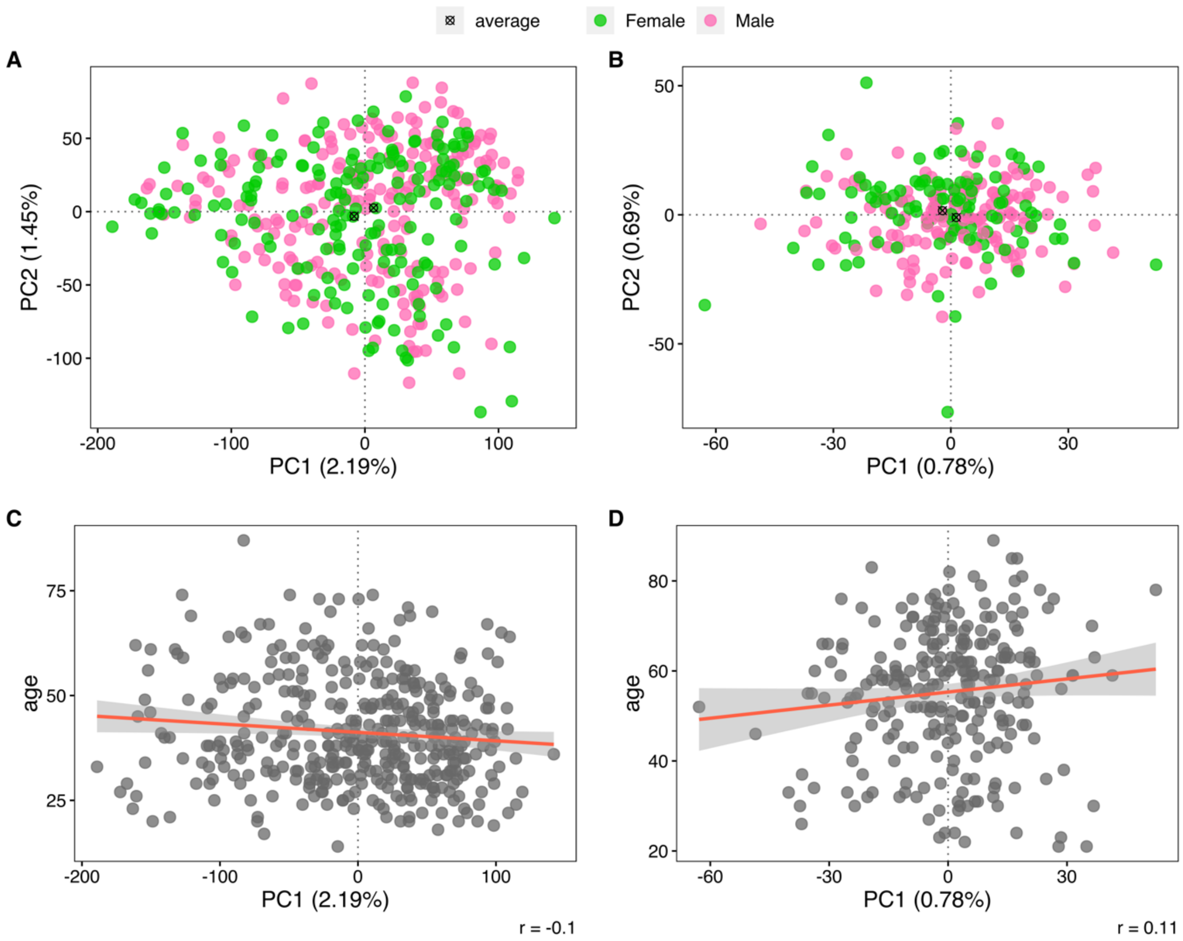

Figure S7. The evaluation of other clinical factors within GM1- and GM2-based data sets. (**A**) PCA for GM1 for LGGs and their sex information. (**B**) PCA for GM2 for LGGs and GBMs and their sex information. (**C**) The variation of the age of LGG samples is based on GM1. (**D**) The variation of the age of LGG and GBM samples is based on GM2. Pearson correlation (r) value is marked in the plot caption.

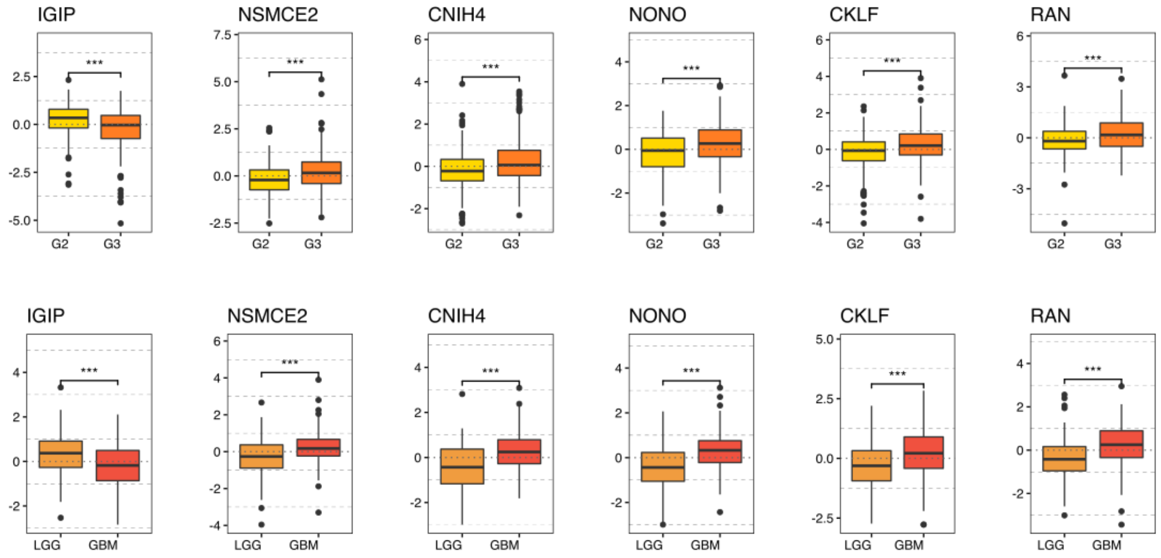

Figure S8. Expression profiles for six common differentially expressed genes (DEGs) were selected based on the intersection of the DEGs list between GII vs. GIII and LGG vs. GBM. Gene expression profiles were generated based on TCGA cohorts. *P* values were marked on boxplots as ns ($P > 0.05$), * ($P \leq 0.05$), ** ($P \leq 0.01$), and *** ($P \leq 0.001$).

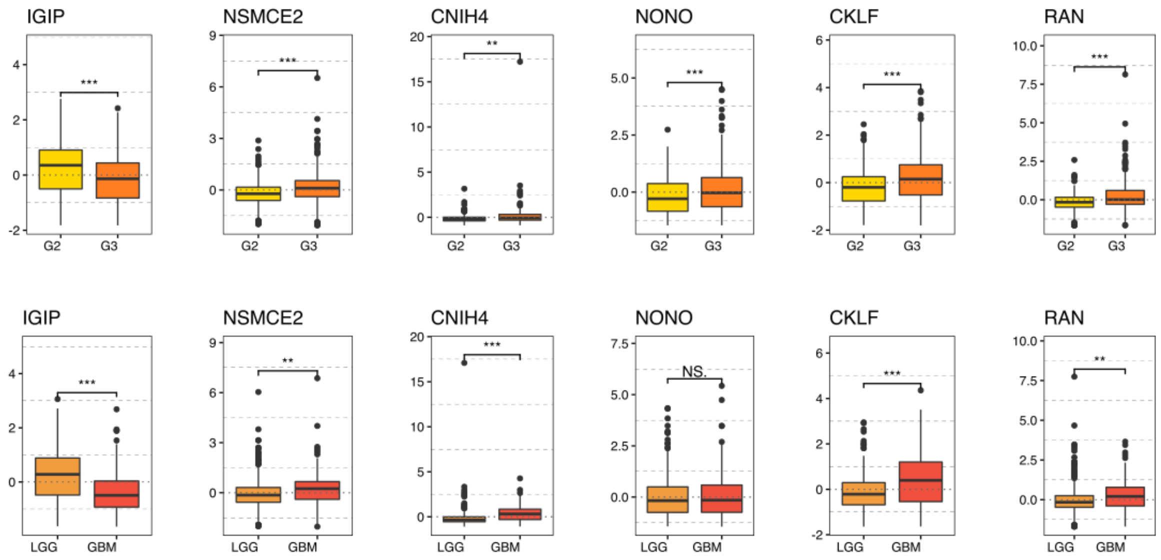

Figure S9. Expression profiles for six common DEGs were selected based on the intersection of DEGs list between GII vs. GIII and LGG vs. GBM. Gene expression profiles were generated based on the Chinese Glioma Genome Atlas (CGGA) batch 1 cohort. *P* values were marked on boxplots as ns ($P > 0.05$), * ($P \leq 0.05$), ** ($P \leq 0.01$), and *** ($P \leq 0.001$).

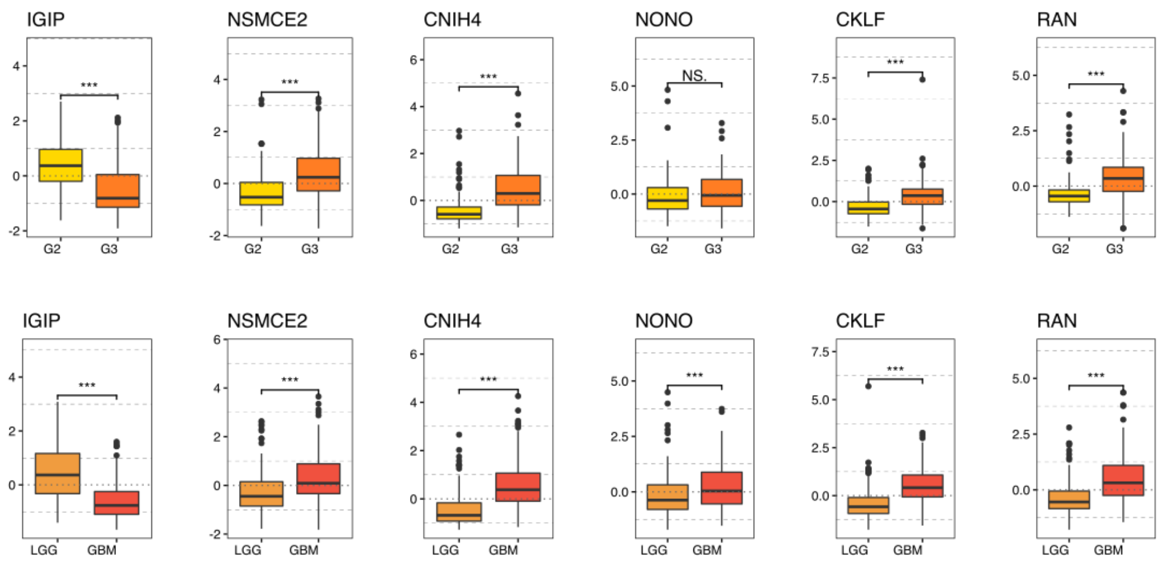

Figure S10. Expression profiles for six common DEGs were selected based on the intersection of DEGs list between GII vs. GIII and LGG vs. GBM. Gene expression profiles were generated based on the CGGA batch 2 cohort. *P* values were marked on boxplots as ns ($P > 0.05$), * ($P \leq 0.05$), ** ($P \leq 0.01$), and *** ($P \leq 0.001$).

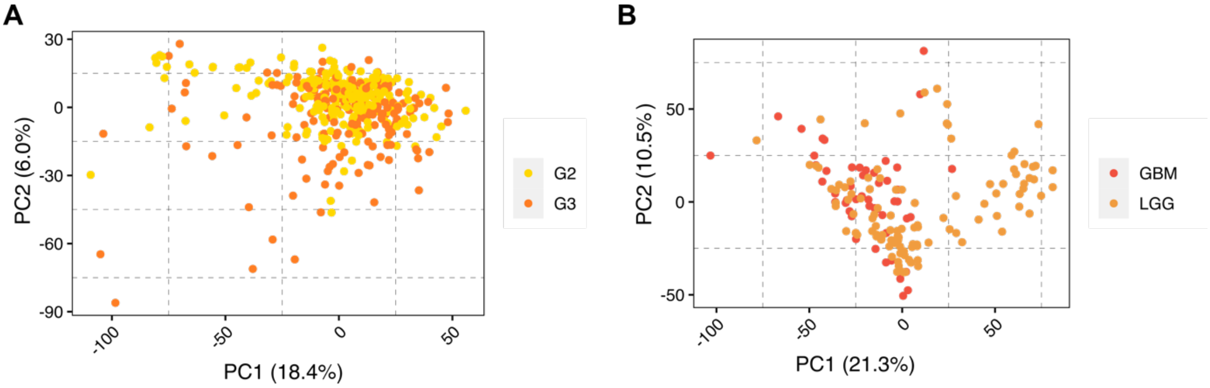

Figure S11. Variability in DNA methylation data from TCGA based on samples selected upon GM modeling for (**A**) GM2 and (**B**) GM1. Explained variation is given in parenthesis.

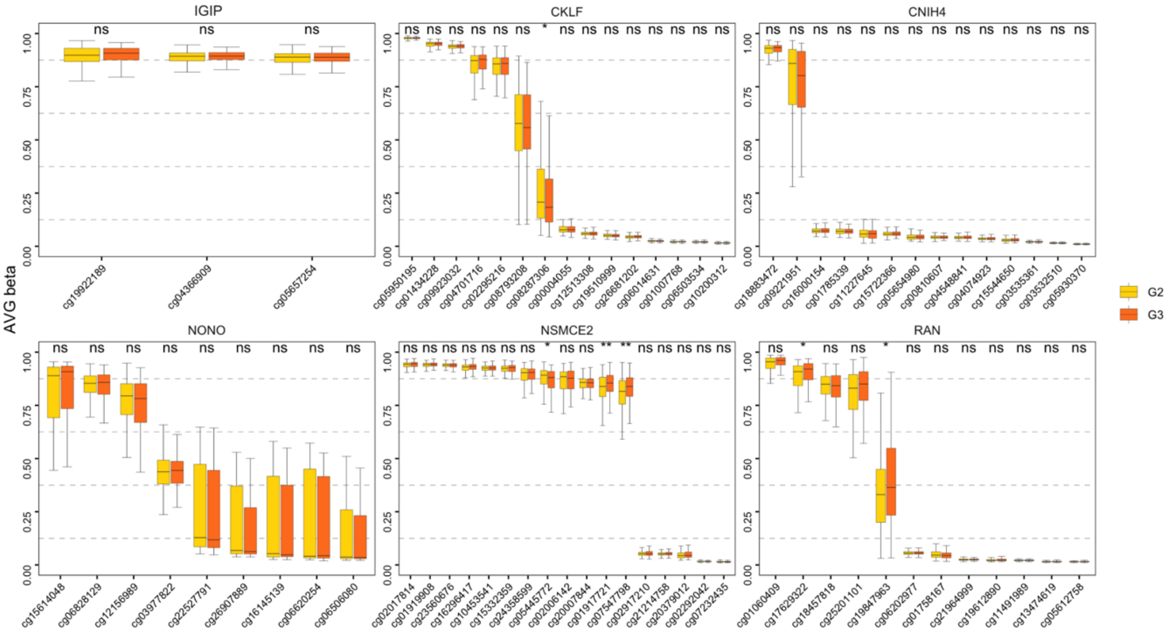

Figure S12. DNA methylation status of six common DEGs for GII vs. GIII. *P* values were marked on boxplots as ns ($P > 0.05$), * ($P \leq 0.05$), ** ($P \leq 0.01$), and *** ($P \leq 0.001$).

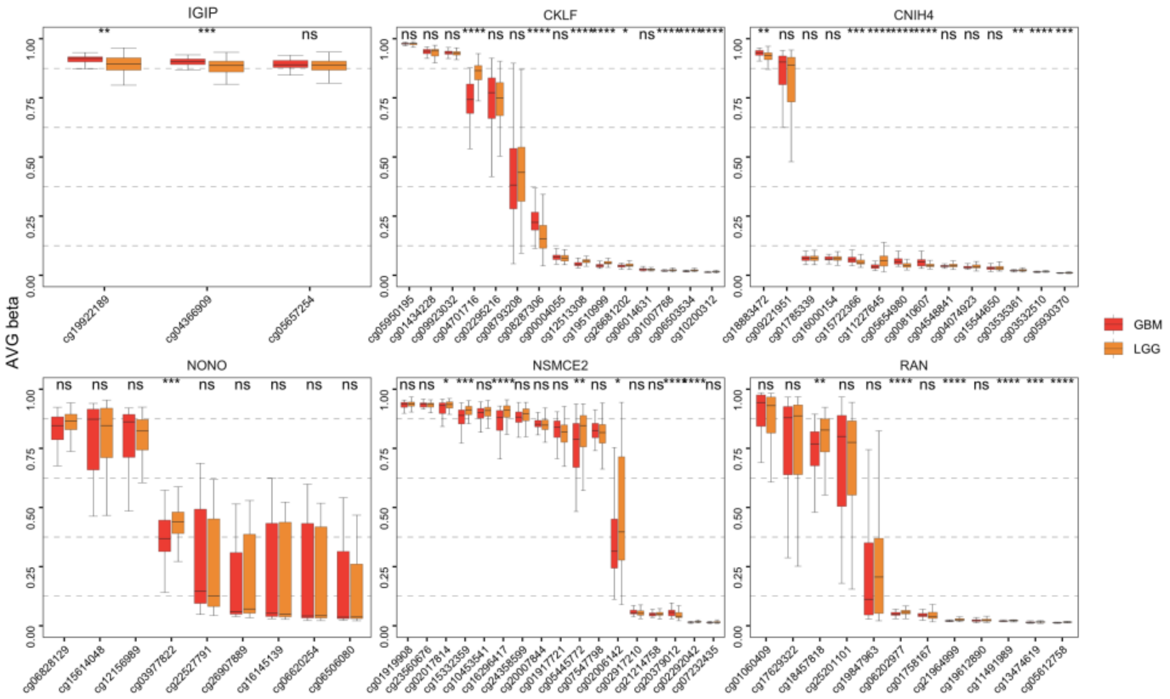

Figure S13. DNA methylation status of six common DEGs for LGG vs. GBM. *P* values were marked on boxplots as ns ($P > 0.05$), * ($P \leq 0.05$), ** ($P \leq 0.01$), and *** ($P \leq 0.001$).

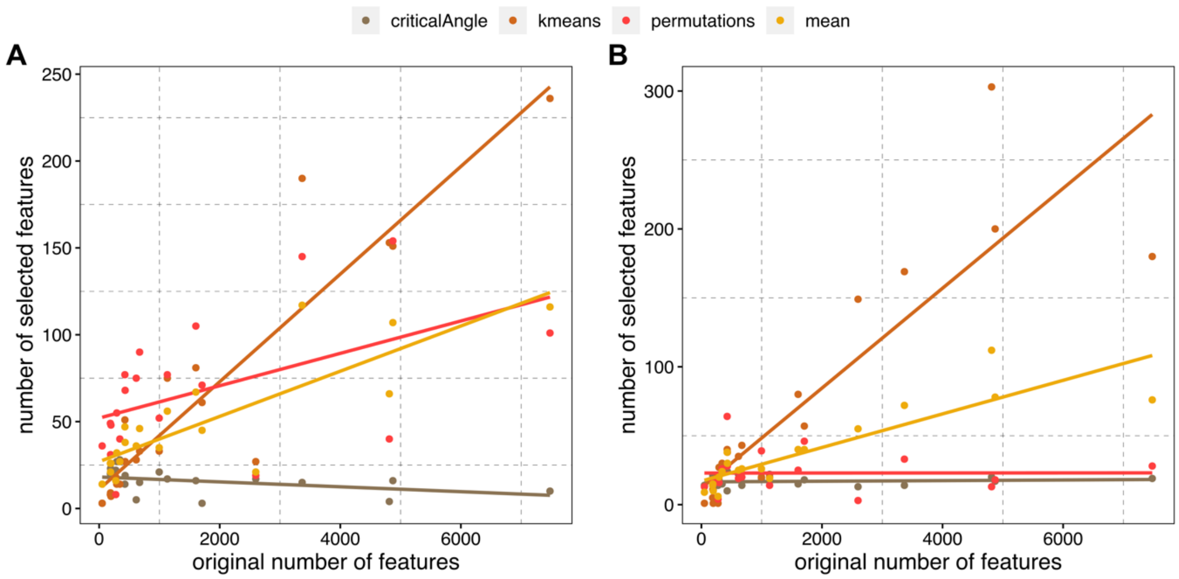

Figure S14. The Monte Carlo feature selection (MCFS) evaluation of thresholds was performed for choosing a threshold for selecting top features. (**A**) All 20 MSigDB collections for GII vs. GIII. (**B**) All 20 MSigDB collections for LGG vs. GBM.

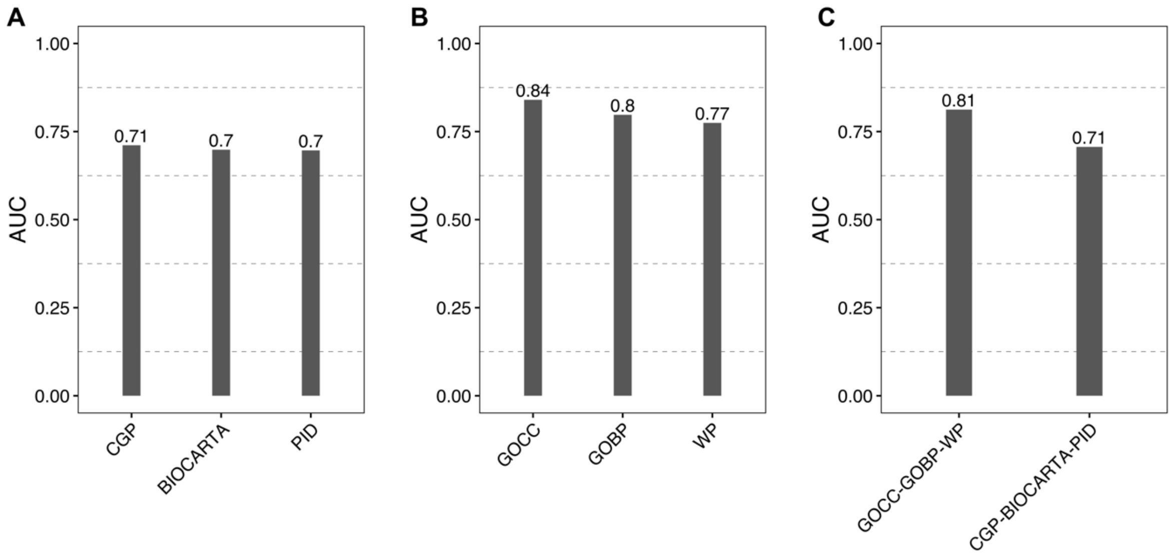

Figure S15. Evaluation of MSigDB collections with R.ROSETTA rule-based learning for classifying glioma grades using ssGSEA scores based on TCGA cohorts. (**A**) Top three MSigDB collections for classifying GII vs. GIII. (**B**) Top three MSigDB collections for classifying LGG vs. GBM. (**C**) Merged top three MSigDB collections for two RBMs classifying GII vs. GIII (right bar), and LGG vs. GBM (left bar).

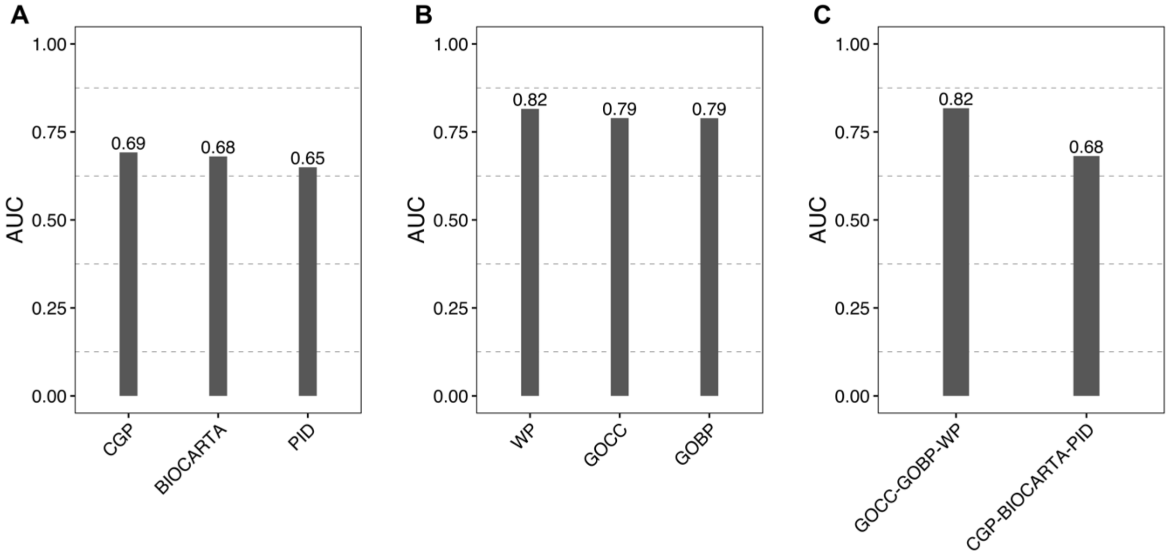

Figure S16. Evaluation of MSigDB collections with R.ROSETTA rule-based learning for classifying glioma grades using ssGSEA scores based on CGGA batch 1. (**A**) Top three MSigDB collections for classifying GII vs. GIII. (**B**) Top three MSigDB collections for classifying LGG vs. GBM. (**C**) Merged top three MSigDB collections for two RBMs classifying GII vs. GIII (right bar), and LGG vs. GBM (left bar).

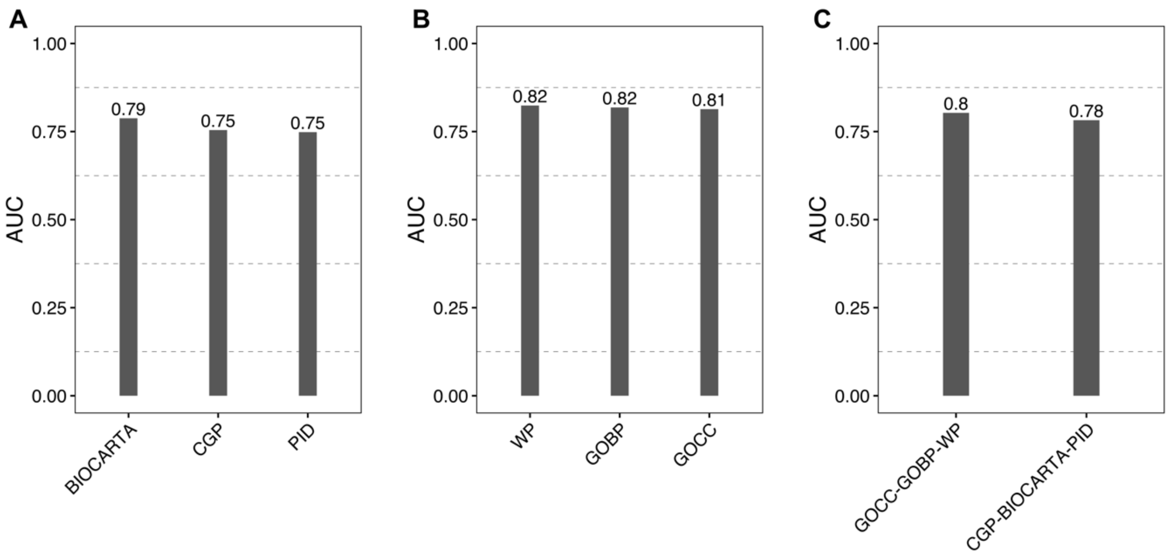

Figure S17. Evaluation of MSigDB collections with R.ROSETTA rule-based learning for classifying glioma grades using ssGSEA scores based on CGGA batch 2. (**A**) Top three MSigDB collections for classifying GII vs. GIII. (**B**) Top three MSigDB collections for classifying LGG vs. GBM. (**C**) Merged top three MSigDB collections for two RBMs classifying GII vs. GIII (right bar), and LGG vs. GBM (left bar).

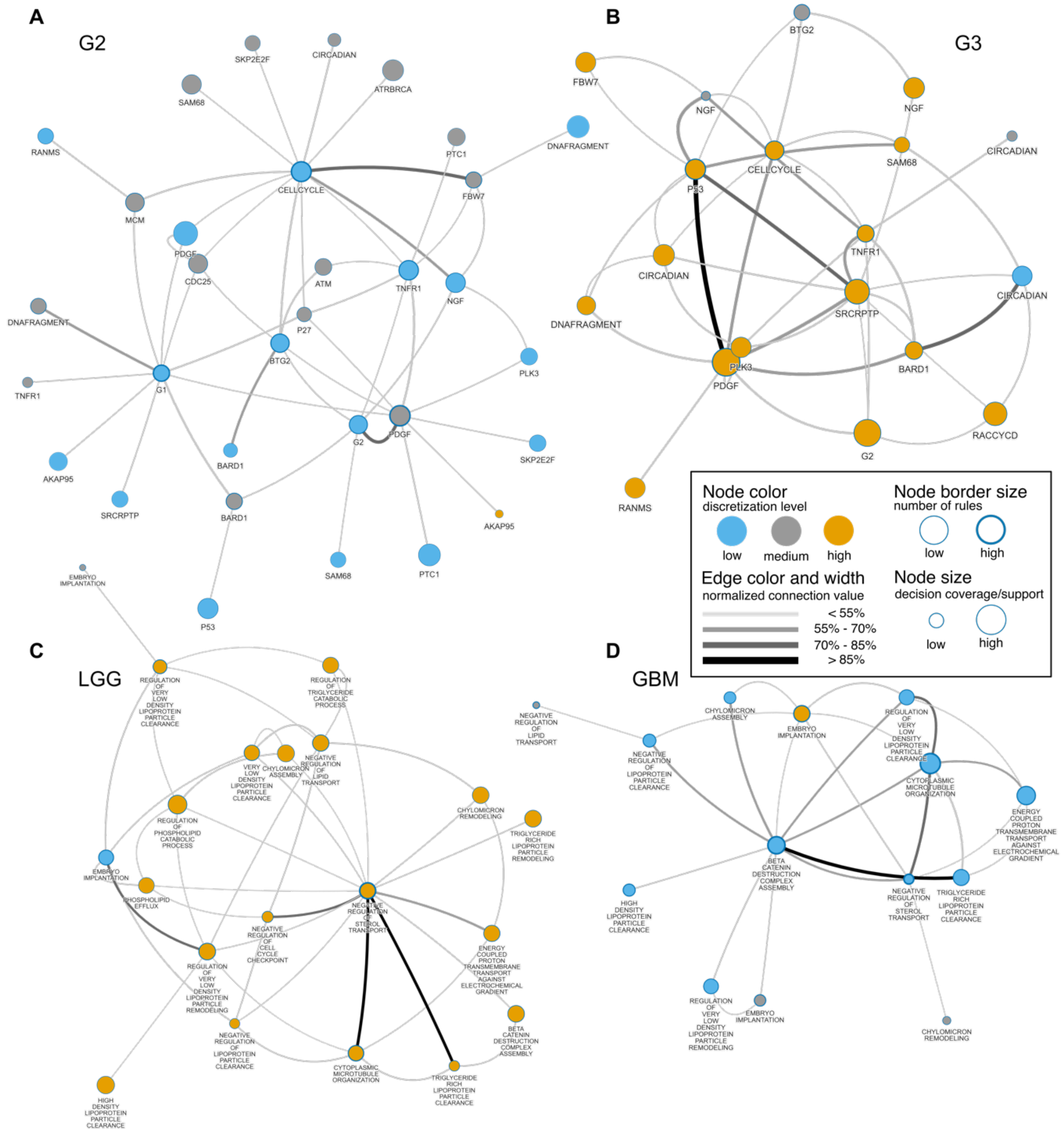

Figure S18. Rule-based network displaying the most relevant co-enrichments of annotations obtained from (**A, B)** the BioCarta collection for the GII vs. GIII model and **(C, D**) the GOBP collection for the LGG vs. GBM model. The networks show 20 most connected nodes obtained from the top 10% based on the rule connection from a set of significant rules (FDR-adjusted *P* value < 0.01). Connection values of nodes and edges represent a strength of co-enrichment from the classifier. Subnetworks were generated separately with respect to the decision class for each RBM.

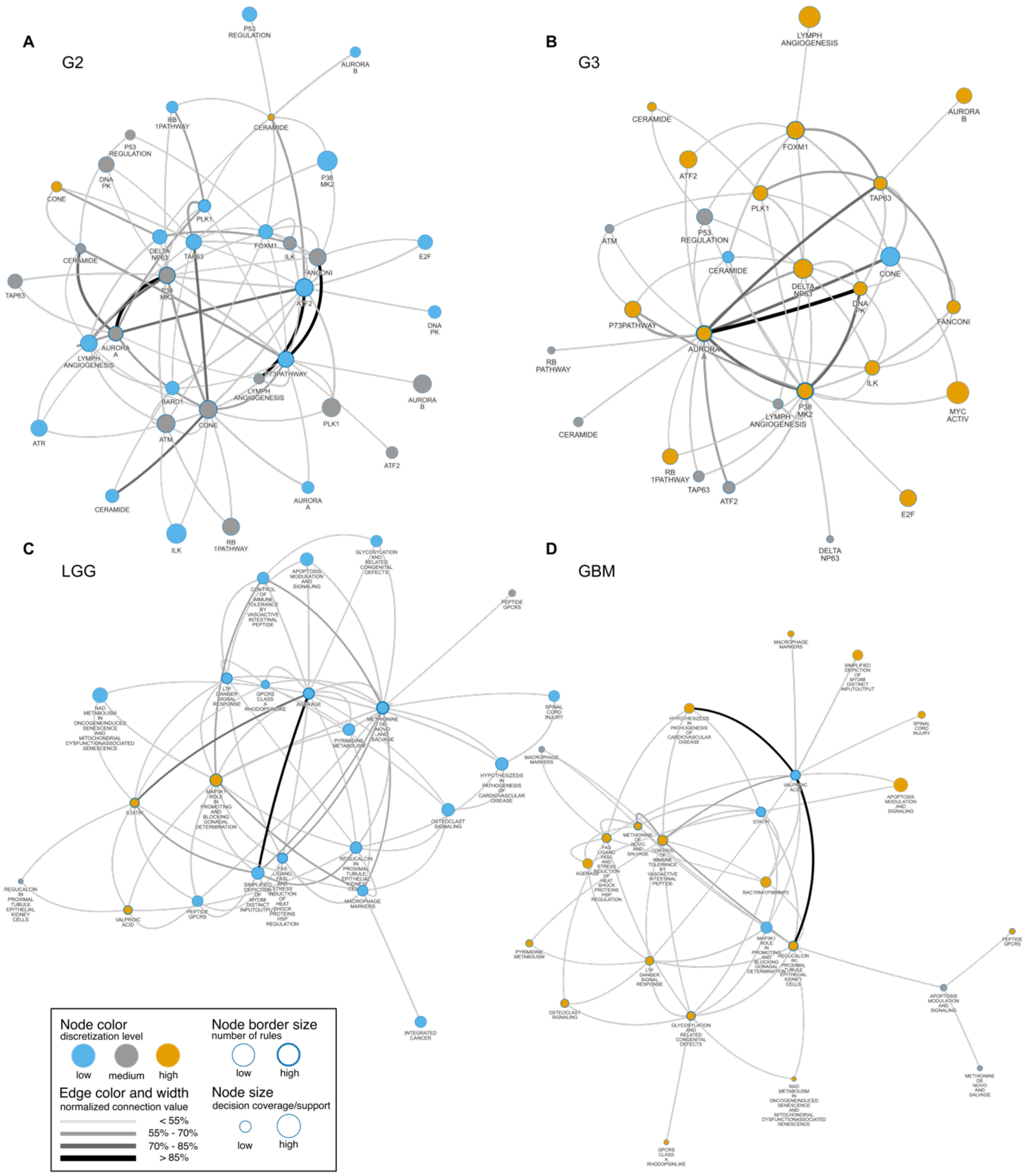

Figure S19. Rule-based network displaying the most relevant co-enrichments of annotations obtained from **(A, B)** PID for the GII vs. GIII model and (**C, D)** WP collections for the LGG vs. GBM model. The networks show 20 most connected nodes obtained from the top 10% based on the rule connection from a set of significant rules (FDR-adjusted *P* value < 0.01). Connection values of nodes and edges represent a strength of co-enrichment from the classifier. Subnetworks were generated separately with respect to the decision class for each RBM.

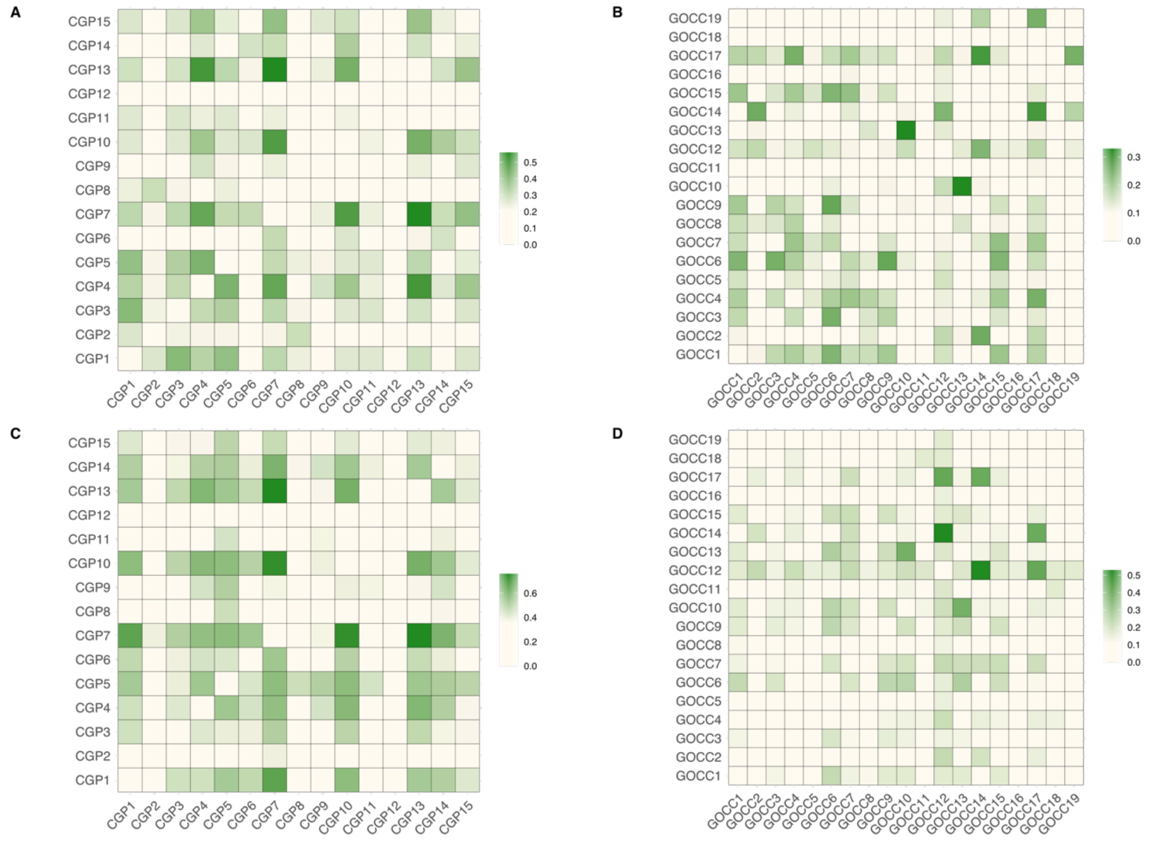

CGP1 HESS_TARGETS_OF_HOXA9_AND_MEIS1_UP
CGP2 MATZUK_SPERMATOCYTE
CGP3 POMEROY_MEDULLOBLASTOMA_PROGNOSIS_DN
CGP4 SHEPARD_BMYB_MORPHOLINO_DN
CGP5 ZAMORA_NOS2_TARGETS_UP
CGP6 MATHEW_FANCONI_ANEMIA_GENES
CGP7 MEBARKI_HCC_PROGENITOR_FZD8CRD_UP
CGP8 PURBEY_TARGETS_OF_CTBP1_AND_SATB1_UP
CGP9 KRIEG_HYPOXIA_NOT_VIA_KDM3A
CGP10 ZERBINI_RESPONSE_TO_SULINDAC_DN
CGP11 MYLLYKANGAS_AMPLIFICATION_HOT_SPOT_15
CGP12 HOLLERN_SOLID_NODULAR_BREAST_TUMOR_UP
CGP13 NAKAYAMA_SOFT_TISSUE_TUMORS_PCA2_UP
CGP14 ZHOU_CELL_CYCLE_GENES_IN_IR_RESPONSE_2HR
CGP15 LI_WILMS_TUMOR_VS_FETAL_KIDNEY_2_UP

GOCC1 INTERMEDIATE_DENSITY_LIPOPROTEIN_PARTICLE
GOCC2 RNA_POLYMERASE_II_CORE_COMPLEX
GOCC3 CHYLOMICRON
GOCC4 SPHERICAL_HIGH_DENSITY_LIPOPROTEIN_PARTICLE
GOCC5 GLYCOSYLPHOSPHATIDYLINOSITOL_N_ACETYLGLUCOSAMINYLTRANSFERASE_GPI_GNT_COMPLEX
GOCC6 LOW_DENSITY_LIPOPROTEIN_PARTICLE
GOCC7 KINESIN_COMPLEX
GOCC8 PYRUVATE_DEHYDROGENASE_COMPLEX
GOCC9 TRIGLYCERIDE_RICH_PLASMA_LIPOPROTEIN_PARTICLE
GOCC10 LYSOSOMAL_LUMEN
GOCC11 RESPIRATORY_CHAIN_COMPLEX_II
GOCC12 ANAPHASE_PROMOTING_COMPLEX
GOCC13 VACUOLE
GOCC14 PROTEIN_DNA_COMPLEX
GOCC15 MICROTUBULE_MINUS_END
GOCC16 HEMIDESMOSOME
GOCC17 MICROTUBULE_ORGANIZING_CENTER
GOCC18 MITOCHONDRIAL_ALPHA_KETOGLUTARATE_DEHYDROGENASE_COMPLEX
GOCC19 LAMELLAR_BODY

Figure S20. Validation of top MSigDB collections: CGP for GII vs. GIII and GOCC for LGG vs. GBM. Heatmaps were generated based on cohort (**A**, **B**) CGGA batch 1 and (**C**, **D**) CGGA batch 2. Values of total correlation among two variables and a decision class given in nats.

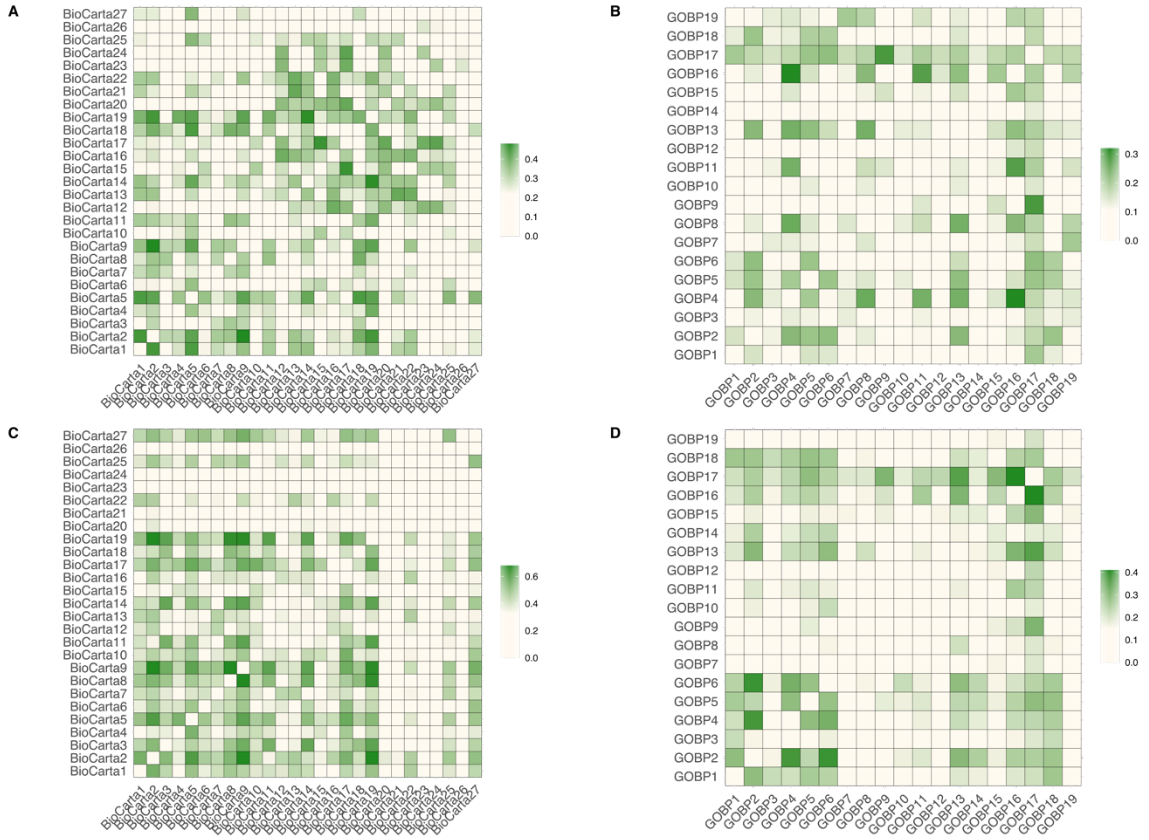

BioCarta1	CELLCYCLE
BioCarta2	G2
BioCarta3	BARD1
BioCarta4	SRCRPTP
BioCarta5	RANMS
BioCarta6	SKP2E2F
BioCarta7	PLK3
BioCarta8	ATRBRCA
BioCarta9	EFP
BioCarta10	FBW7
BioCarta11	CDC25
BioCarta12	TNFR1
BioCarta13	P53
BioCarta14	RB
BioCarta15	SAM68
BioCarta16	G1
BioCarta17	AKAP95
BioCarta18	PTC1
BioCarta19	MCM
BioCarta20	RACCYCD
BioCarta21	BTG2
BioCarta22	ATM
BioCarta23	PDGF
BioCarta24	NGF
BioCarta25	P27
BioCarta26	CIRCADIAN
BioCarta27	DNAFRAGMENT

GOBP1	NEGATIVE_REGULATION_OF_STEROL_TRANSPORT
GOBP2	REGULATION_OF_VERY_LOW_DENSITY_LIPOPROTEIN_PARTICLE_CLEARANCE
GOBP3	TRIGLYCERIDE_RICH_LIPOPROTEIN_PARTICLE_CLEARANCE
GOBP4	REGULATION_OF_VERY_LOW_DENSITY_LIPOPROTEIN_PARTICLE_REMODELING
GOBP5	VERY_LOW_DENSITY_LIPOPROTEIN_PARTICLE_CLEARANCE
GOBP6	NEGATIVE_REGULATION_OF_LIPOPROTEIN_PARTICLE_CLEARANCE
GOBP7	CHYLOMICRON_REMODELING
GOBP8	HIGH_DENSITY_LIPOPROTEIN_PARTICLE_CLEARANCE
GOBP9	NEGATIVE_REGULATION_OF_LIPID_TRANSPORT
GOBP10	PHOSPHOLIPID_EFFLUX
GOBP11	BETA_CATENIN_DESTRUCTION_COMPLEX_ASSEMBLY
GOBP12	ENERGY_COUPLED_PROTON_TRANSMEMBRANE_TRANSPORT_AGAINST_ELECTROCHEMICAL_GRADIENT
GOBP13	CHYLOMICRON_ASSEMBLY
GOBP14	REGULATION_OF_TRIGLYCERIDE_CATABOLIC_PROCESS
GOBP15	NEGATIVE_REGULATION_OF_CELL_CYCLE_CHECKPOINT
GOBP16	CYTOPLASMIC_MICROTUBULE_ORGANIZATION
GOBP17	EMBRYO_IMPLANTATION
GOBP18	REGULATION_OF_PHOSPHOLIPID_CATABOLIC_PROCESS
GOBP19	TRIGLYCERIDE_RICH_LIPOPROTEIN_PARTICLE_REMODELING

Figure S21. Validation of top MSigDB collections: BioCarta pathways for GII vs. GIII and GOBP for LGG vs. GBM. Heatmaps were generated based on cohort (**A**, **B**) CGGA batch 1 and (**C**, **D**) CGGA batch 2. Values of total correlation among two variables and a decision class given in nats.

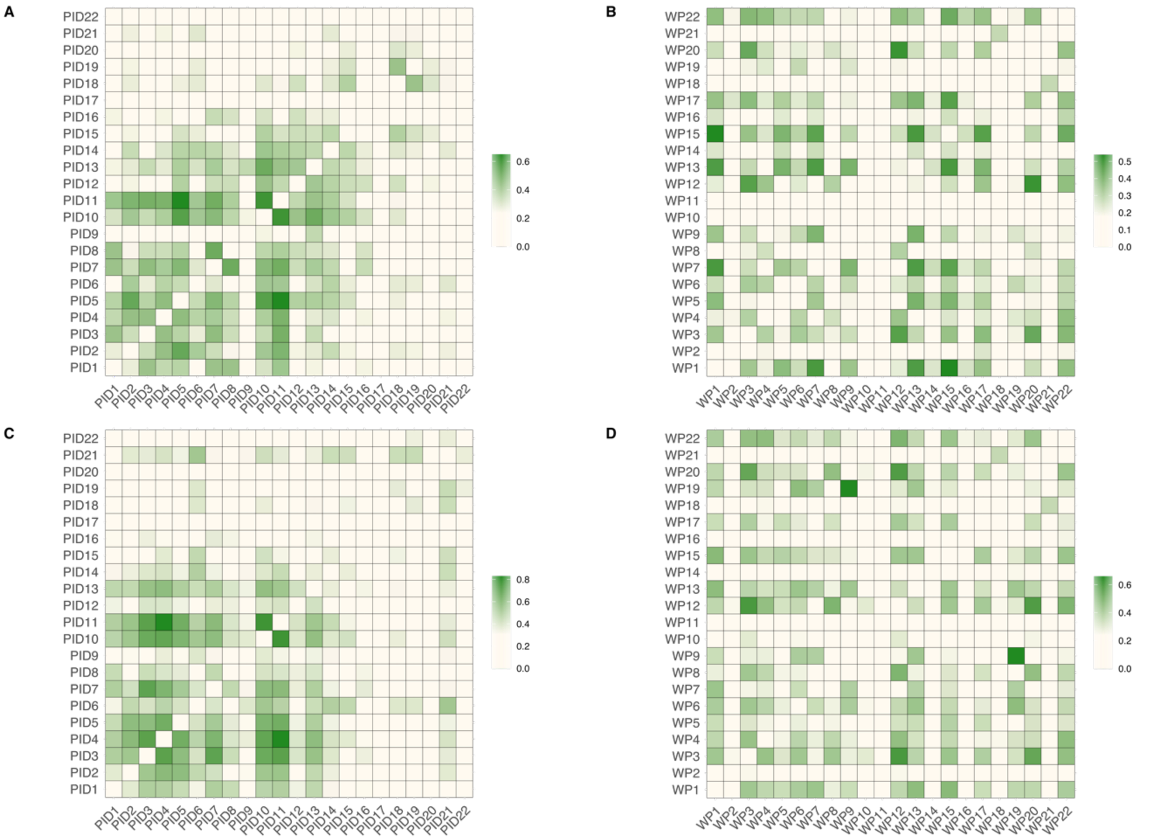

PID1	FANCONI	WP1	HYPOTHESIZED_PATHWAYS_IN_PATHOGENESIS_OF_CARDIOVASCULAR_DISEASE
PID2	AURORA_A	WP2	METHIONINE_DE_NOVO_AND_SALVAGE_PATHWAY
PID3	ATR	WP3	APOPTOSIS_MODULATION_AND_SIGNALING
PID4	FOXM1	WP4	OSTEOCLAST_SIGNALING
PID5	PLK1	WP5	REGUCALCIN_IN_PROXIMAL_TUBULE_EPITHELIAL_KIDNEY_CELLS
PID6	P73PATHWAY	WP6	GLYCOSYLATION_AND_RELATED_CONGENITAL_DEFECTS
PID7	BARD1	WP7	RAC1PAK1P38MMP2_PATHWAY
PID8	ATM	WP8	CONTROL_OF_IMMUNE_TOLERANCE_BY_VASOACTIVE_INTESTINAL_PEPTIDE
PID9	P38_MK2	WP9	INTEGRATED_CANCER_PATHWAY
PID10	E2F	WP10	MAP3K1_ROLE_IN_PROMOTING_AND_BLOCKING_GONADAL_DETERMINATION
PID11	AURORA_B	WP11	STATIN_PATHWAY
PID12	P53_REGULATION	WP12	MACROPHAGE_MARKERS
PID13	MYC_ACTIV	WP13	FAS_LIGAND_FASL_PATHWAY_AND_STRESS_INDUCTION_OF_HEAT_SHOCK_PROTEINS_HSP_REGULATION
PID14	RB_1PATHWAY	WP14	VALPROIC_ACID_PATHWAY
PID15	ILK	WP15	AGERAGE_PATHWAY
PID16	DNA_PK	WP16	NAD_METABOLISM_IN_ONCOGENEINDUCED_SENESCENCE_AND_MITOCHONDRIAL_DYSFUNCTIONASSOCIATED_SENESCENCE
PID17	CONE	WP17	SIMPLIFIED_DEPICTION_OF_MYD88_DISTINCT_INPUTOUTPUT_PATHWAY
PID18	LYMPH_ANGIOGENESIS	WP18	PEPTIDE_GPCRS
PID19	ATF2	WP19	PYRIMIDINE_METABOLISM
PID20	CERAMIDE	WP20	LTF_DANGER_SIGNAL_RESPONSE_PATHWAY
PID21	DELTA_NP63	WP21	GPCRS_CLASS_A_RHODOPSINLIKE
PID22	TAP63	WP22	SPINAL_CORD_INJURY

Figure S22. Validation of top MSigDB collections: PID for GII vs. GIII and WP for LGG vs. GBM. Heatmaps were generated based on cohort (**A**, **B**) CGGA batch 1 and (**C**, **D**) CGGA batch 2. Values of total correlation among two variables and a decision class given in nats.

Supplementary Captions for Tables S1-S8

Table S1. Computational methods and R packages that were used in this study.
Table S2. GM1 and GM2 subsets retrieved from GM modelling of PC1.
Table S3. A list of significant DEGs for G2 vs. G3 (FDR < 0.001).
Table S4. A list of significant DEGs for LGG vs. GBM (FDR < 0.001).
Table S5. Significant enrichment results (FDR < 0.05) for G2 vs. G3 DEGs from *gProfiler*.
Table S6. Significant enrichment results (FDR < 0.05) for LGG vs. GBM DEGs from *gProfiler*.
Table S7. A list of significant rules (FDR < 0.01) for G2 vs. G3 RBM built for the CGP collection.
Table S8. A list of significant rules (FDR < 0.01) for LGG vs. GBM RBM built for the GOCC collection.

Supplementary References for Table S1

Draminski, M., & Koronacki, J. (2018). rmcfs: An R Package for Monte Carlo Feature Selection and Interdependency Discovery. *Journal of Statistical Software*, *85*, 1–28. https://doi.org/10.18637/JSS.V085.I12

Garbulowski, M., Diamanti, K., Smolińska, K., Baltzer, N., Stoll, P., Bornelöv, S., Øhrn, A., Feuk, L., & Komorowski, J. (2021). R.ROSETTA: an interpretable machine learning framework. *BMC Bioinformatics*, *22*(1), 1–18. https://doi.org/10.1186/S12859-021-04049-Z/FIGURES/3

Gentleman, R., Carey VJ, Huber W, & Hahne F. (2021). *genefilter: genefilter: methods for filtering s*https://bioconductor.org/packages/release/bioc/html/genefilter.html

Hansen, K. D. (2016). IlluminaHumanMethylation450kanno. ilmn12. hg19: Annotation for Illumina's 450k methylation arrays. *R Package Version 0.6. 0*, *10*, B9.

Hänzelmann, S., Castelo, R., & Guinney, J. (2013). GSVA: Gene set variation analysis for microarray and RNA-Seq data. *BMC Bioinformatics*, *14*(1), 1–15. https://doi.org/10.1186/1471-2105-14-7/FIGURES/7

Hornik, K., Buchta, C., & Zeileis, A. (2008). Open-source machine learning: R meets Weka. *Computational Statistics 2008 24:2*, *24*(2), 225–232. https://doi.org/10.1007/S00180-008-0119-7

John, C. R., Watson, D., Russ, D., Goldmann, K., Ehrenstein, M., Pitzalis, C., Lewis, M., & Barnes, M. (2020). M3C: Monte Carlo reference-based consensus clustering. *Scientific Reports 2020 10:1*, *10*(1), 1–14. https://doi.org/10.1038/s41598-020-58766-1

Kassambara, A., Kosinski, M., Biecek, P., & Fabian, S. (2021). Package 'survminer'-drawing survival curves using 'ggplot2.' *R Cran*.

Leek, J. T., Johnson, W. E., Parker, H. S., Jaffe, A. E., & Storey, J. D. (2012). The sva package for removing batch effects and other unwanted variation in high-throughput experiments. *Bioinformatics*, *28*(6), 882. https://doi.org/10.1093/BIOINFORMATICS/BTS034

Meyer, P. E., & Meyer, M. P. E. (2009). Package 'infotheo.' *R Package Version; Citeseer: Princeton, NJ, USA*, *1*.

Scrucca, L., Fop, M., Murphy, T. B., & Raftery, A. E. (2016). mclust 5: Clustering, Classification and Density Estimation Using Gaussian Finite Mixture Models. *The R Journal*, *8*(1), 289. https://doi.org/10.32614/rj-2016-021

Smyth, G. K. (2005). Limma: linear models for microarray data. In *Bioinformatics and computational biology solutions using R and Bioconductor* (pp. 397–420). Springer.

Therneau, T. M., & Lumley, T. (2015). Package 'survival.' *R Top Doc*, *128*(10), 28–33.

Vu, V. Q. (2011). ggbiplot: A ggplot2 based biplot. *R Package Version 0.55*, *755*.
